# Survey of *Naegleria fowleri* in Geothermal Recreational Waters of Guadeloupe (French West Indies)

**DOI:** 10.1371/journal.pone.0054414

**Published:** 2013-01-18

**Authors:** Mirna Moussa, Johan F. De Jonckheere, Jérôme Guerlotté, Vincent Richard, Alexandra Bastaraud, Marc Romana, Antoine Talarmin

**Affiliations:** 1 Unité Environnement-Santé, Institut Pasteur de la Guadeloupe, Les Abymes, Guadeloupe, France; 2 De Duve Institute, Brussels, Belgium; 3 Scientific Institute of Public Health, Brussels, Belgium; 4 Université des Antilles et de la Guyane, Pointe à Pitre, Guadeloupe, France; 5 Muséum National d’Histoire Naturelle, UMR 7205 MNHN/CNRS, Paris, France; 6 Institut Pasteur de Dakar, Dakar, Senegal; 7 Inserm, U 665, Pointe-à-Pitre, Guadeloupe, France; Centro de Investigacion y de Estudios Avanzados del Instituto Politecnico Nacional, Mexico

## Abstract

In 2008 a fatal case of primary amoebic meningoencephalitis, due to the amoeboflagellate *Naegleria fowleri*, occurred in Guadeloupe, French West Indies, after a child swam in a bath fed with geothermal water. In order to improve the knowledge on free-living amoebae in this tropical part of France, we investigated on a monthly basis, the presence of *Naegleria* spp. in the recreational baths, and stream waters which feed them. A total of 73 water samples, 48 sediments and 54 swabs samples were collected from 6 sampling points between June 2011 and July 2012. The water samples were filtered and the filters transferred to non-nutrient agar plates seeded with a heat-killed suspension of *Escherichia coli* while sediment and swab samples were placed directly on these plates. The plates were incubated at 44°C for the selective isolation of thermophilic *Naegleria*. To identify the *Naegleria* isolates the internal transcribed spacers, including the 5.8S rDNA, were amplified by polymerase chain reaction and the sequence of the PCR products was determined. Thermophilic amoebae were present at nearly all collection sites. The pathogenic *N. fowleri* was the most frequently encountered thermophilic species followed by *N. lovaniensis*. The concentration of *N. fowleri* was rather low in most water samples, ranging from 0 to 22 per liter. Sequencing revealed that all *N. fowleri* isolates belonged to a common Euro-American genotype, the same as detected in the human case in Guadeloupe. These investigations need to be continued in order to counsel the health authorities about prevention measures, because these recreational thermal baths are used daily by local people and tourists.

## Introduction

Free-living amoebae (FLA) are highly diverse and show a worldwide distribution in aquatic habitats and soils. *Naegleria* is a free-living amoeboflagellate found in freshwater and feeding on bacteria. One species, *N. fowleri*, causes primary amoebic meningoencephalitis (PAM) in man [1], a rare but rapidly fatal central nervous system disease occurring after exposure to contaminated water. Phagocytic FLA are also carriers of potentially pathogenic microorganisms (e.g. *Legionella*) and as such, present an extra threat to human health [2]. FLA have been isolated from natural and man-made aquatic habitats such as fountains, swimming pools and spas, industrial cooling circuits and even from tap water [3]–[5]. Earlier studies indicated that densities of thermophilic amoebae in general and of thermophilic *Naegleria* spp. in particular correlated positively with higher temperatures of naturally or artificially heated waters [6]–[9]. Within the genus, *N. fowleri* as well as other ecological competitors, such as *N. lovaniensis*, tolerate temperatures of up to 45°C and proliferate successfully in natural bodies of water (e.g. lakes and rivers) during warmer months of the year, as well as in geothermally heated water or in industrial cooling water [10]. Such species which can survive at temperatures of 37°C and higher [11] are thermotolerant competitors that develop better than *N. fowleri* at these temperatures [12]. While there are more than 40 species of *Naegleria* described [5], only one species, *N. fowleri* causes PAM. The disease occurs in otherwise healthy individuals with exposure to warm, untreated or poorly disinfected water with chlorine concentrations lower than 1 mg/l [13]–[15]. It is generally acquired while swimming and diving in freshwater lakes and ponds [16], [17]. Infection of the brain occurs after amoebae reach the nasal cavity and invade the nasal mucosa. From there the amoebae penetrate the nasal epithelium to the olfactory nerves and migrate through the cribriform plate to invade the brain and meninges [18]. In April 2008, a 9-year-old previously healthy boy was referred to the Pointe-à-Pitre University Hospital of Guadeloupe (French West Indies) with a 2-day history of high-grade fever, severe headache, and seizures [19]. The possibility of a free-living amoeba infection was suspected both on the negativity of all bacterial and viral initial tests and on the observation of peculiar cells in stained cerebrospinal fluid samples, and was subsequently confirmed to be a PAM case by molecular methods. This first case of PAM in Guadeloupe was rapidly fatal in 7 days. There are many hot springs around the Soufrière volcano on the Basse-Terre part of Guadeloupe, and the patient presumably acquired the infection by swimming and diving in the Dolé bath supplied by natural thermal water one week before onset of the disease.

Guadeloupean people frequently come in contact with raw water at recreational springs, and visiting hot springs is a very popular form of recreation. The water sources of the hot baths are geothermal springs and streams. However, the potential infestation of *N. fowleri* in recreational waters has not been studied yet in Guadeloupe. An investigation of the occurrence and distribution of *Naegleria* in local water was imperative because of its possible health implications, as demonstrated by the PAM case [19]. During a few months at the beginning of 2011, we sampled a number of hot waters of Guadeloupe to better understand the importance of the contamination of these waters. We observed that thermophilic amoeba and especially *Naegleria fowleri* were often detected although at low concentrations. We therefore undertook the following study to investigate the distribution and the density of thermophilic FLA in the most frequented recreational areas of Guadeloupe in order to evaluate the risk for humans and inform the health authorities.

## Materials and Methods

### Sample Collection

**Figure 1:**
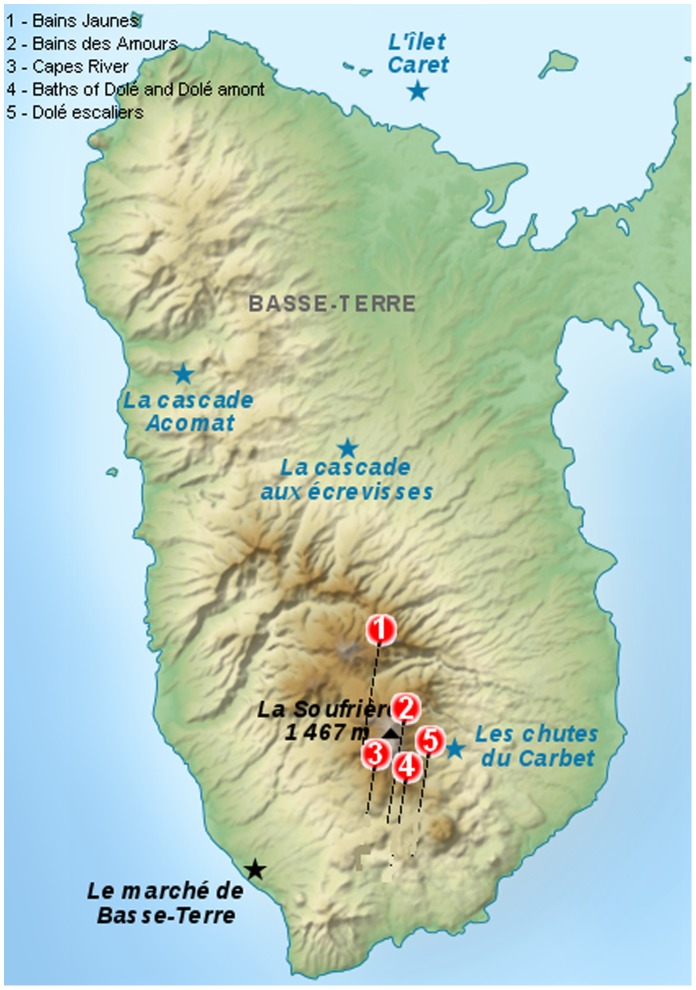
Geographical distribution of sampling sites in the hot waters of the Basse-Terre of Guadeloupe. (1) the Bains Jaunes on the volcano near Saint Claude, (2) the Bains des Amours, (3) Capes River, (4) the bath of Dolé and Dolé Amont and (5) Dolé Escalier in Gourbeyre.

In Guadeloupe, a total of thirty geothermal resurgences can be counted, all of them being located on the island of Basse-Terre. Six of these, the most frequented by the population and tourists, and located around the Soufrière volcano in the area of Gourbeyre and Saint-Claude (Fig. 1), were investigated in our study. Bains Jaunes, Bain de Dolé and Bains des Amours are recreational baths regularly washed with detergent and treated with chlorine by the staff in charge of maintenance. Dolé Amont and Capes are rivers. Dolé Escalier is an old geothermal baths nowadays untreated. Water samples were collected in these places between June 2011 and July 2012. Most frequented baths were analysed monthly, while more remote areas were less regularly tested, depending on weather conditions. Temperature and pH were measured on site for each sample taken. From each sampling point, one to four water samples were collected by submerging 500 ml sterile bottles beneath the surface of the water as well as at the bottom. Swabs (VWR, Strasbourg France) were gently scraped on around 10 cm^2^ of rocks and interiors of baths to collect biofilm from each of these waterbodies as previously described [20], [21]. When possible sediments were also taken in 15 ml sterile tubes. The water samples, all above 28°C, were transported to the laboratory at ambient temperature (around 31°C in Guadeloupe) to maintain the thermophilic amoebae in more natural conditions and were processed within 2 to 4 h after sampling.

### Chemical Parameters

Besides pH and temperature, some chemical parameters were measured in water samples taken from the most frequented baths from October 2011 to January 2012. These parameters were measured in a lab accredited by the French committee for Accreditation (COFRAC), according to French or international normalized methods. Turbidity by nephelometric determination [22]; alcalinity by potentiometric determination [23], hardness by potentiometric determination [24]; K, Ca, Mg, Na by ionic chromatography [25]; SO_4_, Cl by ionic chromatography [26]; SiO_2_ by molecular absorption spectrometry [27]; total organic carbon by combustion and IR spectrometry (TOC analyzer OI Aurora 1030) [28]; permanganate oxidizability [29]; NH4 by continuous flow analysis [30]; NO_3_ by continuous flow analysis [31]. HCO_3_ was calculated according to Legrand & Poirier method [32].

### Isolation of Amoebae

The water samples were thoroughly mixed and the total sample volume (500 ml) was filtered through a cellulose nitrate filter (pore size, 1.2 µm; diameter, 47 mm, Millipore (Fisher Scientific) [33], [34]. Each filter was cut into ten pieces, and placed inverted diagonally on two 1.5% non-nutrient agar plates seeded with a thin layer of *Escherichia coli* (NNA-*E. coli*) [35] (*i.e*., five pieces of filter per plate). Cotton wool swap samples were directly streaked in the middle of the NNA- *E. coli* plates. One or two drops of the sediment samples were placed directly on NNA-*E. coli* plates. Plates were sealed with parafilm (Parafilm ‘M’®) and incubated inverted at 44°C to select for thermophilic amoebae. The plates were inspected daily for amoebic growth (appearance of clearing zones) for up to 7 days.

### Morphological Identification and Amoeba Counting

During incubation, the first line plates were examined with an inverted phase contrast microscopy (Will-Wetzlar “Wilovert” x400). Every amoeba plaque emerging along the filters was counted and picked for isolation and subcultured, on fresh NNA-*E. coli* (second line plates). The number of amoeba was multiplied by 2 to obtain a number/liter if only 500 ml had been filtered or a mean was realized if 2 or more 500 ml samples were filtered. Above 2 plaques per piece of filter, we considered that the precision was too low and therefore indicated the concentration of amoebae as ≥40/l. The amoebic isolates were identified morphologically using Page’s taxonomy keys [35]. The definitive identification was systematically realized by PCR.

### Statistical Analysis

Data were analysed using R software. The results were expressed as median and mean. Fisher’s exact test was used to study categorical variables while the Kruskall Wallis test and the Spearman test were used to study non-parametric variables. Because of the lack of precision above 20 amoebae/liter data were analysed using classes (absence, <10/l, ≥10 and <20/l and ≥20/l). Values were considered statistically significant for p<0.05.

### DNA Extraction

Amoebae from second line plate were flushed from the agarose with 200 to 500 µl of UNSET [36] lysis solution (urea 8 M, SDS 2%, NaCl 0.15 M, EDTA 0.001 M, Tris pH 7.5 0.1 M) and transferred into 1.5 ml tubes. DNA was further purified using a commercial kit (Qiagen DNA Mini kit. Qiagen, France) following the manufacturer’s instructions and stored at −20°C until further processing.

### PCR Amplification and Sequencing

The complete ITS region (ITS1, 5.8S, and ITS2) was amplified using the primers previously described [37], [38]. The ITS species specific primers were for *N. fowleri* : the forward primer NFITSFW, (5′-TGAAAACCTTTTTTCCATTTACA-3′) and the reverse primer NFITSRV (5′-AATAAAAGATTGACCATTTGAAA-3′), for *Naegleria* sp : the forward primer ITSFW (5′-AACCTGCGTAGGGATCATTT -3′) and the reverse primer ITSRV (5′-TTTCCTCCCCTTATTAATAT-3′) and for other *Vahlkamphfiidae* : the forward primer JITSFW (5′-GTCTTCGTAGGTGAACCTGC-3′) and the reverse primer JITSRV (5′-CCGCTTACTGATATGCTTAA- 3′). Amplification was carried out with a PCR mix (5X Green or Colorless GoTaq Flexi Buffer, Promega) containing 2.5 mM MgCl_2_ and 0.25 mM of each dNTP, 1 µM of each primer, 1.25 U of Taq DNA polymerase, and 50–100 ng of genomic DNA in a final volume of 50 µl. Amplifications were run in a BioRad thermocycler. After 6 min for denaturation at 94°C, the PCR temperature profiles consisted of 30/40 cycles at 94°C for 1 min, 55°C for 1.5 min, and 72°C for 2 min with a final elongation step at 72°C for 10 min.

Aliquots (10 µl) of each PCR products were mixed with 2 µl of loading buffer (10 mM EDTA, 10% glycerol, 0.015% bromophenol blue, 0.17% SDS), run in a 1% agarose gel in TBE buffer (89 mM Tris, 89 mM Boric Acid, 2 mM EDTA, pH 8.3) and visualized with ethidium bromide. A 100-bp DNA ladder (Invitrogen) was used as a size marker in the gels. Negative DNA controls (template DNA replaced with distilled water), and positive controls (*N. fowleri* # 0359) and sample DNA were analyzed using the three sets of primers.

### Nucleotide Sequencing

The PCR products obtained with the ITS primers were purified using a QIAamp PCR purification kit (Qiagen) and directly sequenced in both directions with the respective forward and reverse primers using the BigDye Terminator Sequencing v3.1 kit and an ABI 310™ Genetic Analyzer (Applied Biosystems, Foster City, CA, USA). A homology search was performed with BLAST software from the National Center for Biotechnology Information homepage (http://www.ncbi.nlm.nih.gov/). The sequence data obtained were aligned by ClustalW software (http://www.nig.ac.jp) with the sequences of *Naegleria* species deposited in GenBank (http://www.ncbi.nlm.nih.gov/).

### Nucleotide Sequence Accession Numbers

The nucleotide sequences determined from this study were submitted to GenBank and assigned the accession numbers JX910445, JX910446, JX910447.

### Ethic Statement

All baths tested are public baths that are managed by towns or villages. All necessary permits were obtained for the described field studies. These permits were given by Regional Health Agency (ARS) which has the responsibility for testing the quality of all kind of waters in Guadeloupe (for drinking or bathing).

The field studies did not involve endangered or protected species.

## Results

The results of the monthly monitoring for thermophilic amoebae occurrence are presented in tables 1 and 2. Thermophilic amoebae were detected at least one time at all collection sites, in 35 out of the 73 water samples analyzed (47.9%), 18 of the 48 sediment samples (37.5%) and 8 of 54 swabs tested (14.8%) (p<0.01). *N. fowleri* was the most frequently encountered thermophilic species, found in 28 of the 73 water samples (38.3%), 5 of the 48 sediment samples (10.4%) and none of the 54 swab samples collected (p<0.01). *N. lovaniensis* was less frequently isolated than *N. fowleri.* It was found in 7 of the 73 water samples (9.6%), 5 of the 48 sediment samples (10.4%) and in none of the 54 swab samples collected (p<0.01).

**Table 1:** Survey of free-living amoeba isolated from geothermal recreational waters of Guadeloupe on a montly basis (2011).

Baths		Average		June			July			August			September			October			November			December		
		T	pH	Sp.n	*N.l* n	*N.f* n	Sp n.	*N.l* n	*N.f* n	Sp. n	*N.l* n	*N.f* n	Sp.n	*N.l* n	*N.f*n	Sp.n	*N.l* n	*N.f* n	Sp. n	*N.l* n	*N.f*n	Sp. n	*N.l* n	*N.f* n
**Bains**	Water	31,5	6,3	0	0	0	0	0	0	0	0	0	0	0	2	0	0	5	1	0	10	0	0	0
**Jaunes**	Sed			-	-	-	-	-	-	-	-	-	-	-	-	-	-	-	-	-	-	-	-	-
	Swab			-	-	-	-	-	-	-	-	-	-	-	-	-	-	-	-	-	-	-	-	-
**Bain**	Water	34,4	7,2	0	0	0	0	0	0	0	0	1	0	0	0	0	0	0	0	0	0	0	0	0
**des**	Sed			-	-	-	-	-	-				-	-	-	-	-	-	-	-	-			
**Amours**	Swab			-	-	-	-	-	-							-	-	-	-	-	-	-	-	-
**Bain de**	Water	29.8	7,2	9	0	0										4	0	6				6	4	0
**Capès**	Sed			+	-	-										-	-	-				-	-	-
	Swab															-	-	-				-	-	-
**Bain de**	Water	31,6	8,3	0	0	8	0	20	8	0	0	7	0	0	6	0	0	2	1	0	13	0	0	6
**Dolé**	Sed																							
	Swab			-	-	-	-	-	-	-	-	-	-	-	-	-	-	-	-	-	-	-	-	-
**Dolé**	Water	31,6	7,5				0	0	22	0	0	0	0	2	2	0	0	6	0	0	2	0	0	9
**Amont**	Sed						-	-	-							-	-	-						
	Swab																		-	-	-	+	-	-
**Dolé**	Water	30,5	7,1	0	0	0																0	0	0
**Escalier**	Sed																							
	Swab																							

**Sp. :** thermophilic amoebae non-Naegleria sp., **N.l** : Naegleria lovaniensis., **N.f** : Naegleria fowleri,

(n) number of amoebae per liter of water; (+ ou -) presence or absence in sediment or swab; (blank) not investigated.

**Table 2:** Survey of free-living amoeba isolated from geothermal recreational waters of Guadeloupe on a montly basis (2012).

**Baths**		**Average**		**January**			**February**			**March**			**April**			**May**			**June**			**July**		
		**T**	**pH**	Sp. n	*N.l* n	*N.f* n	Sp.n	*N.l* n	*N.f* n	Sp. n	*N.l* n	*N.f* n	Sp. n	*N.l* n	*N.f* n	Sp.n	*N.l* n	*N.f* n	Sp. n	*N.l* n	*N.f* n	Sp. n	*N.l* n	*N.f* n
**Bains**	Water	31,3	6,7	0	0	0	0	0	0	0	0	0	0	0	0	0	0	0	0	0	3	0	0	0
**Jaunes**	Sed						−	−	−	−	−	−	−	−	−	−	−	−	−	−	−	−	−	−
	Swab			−	−	−				−	−	−	+	−	−	−	−	−	−	−	−	−	−	−
**Bain**	Water	34,1	7,3	0	0	0	0	0	0	0	0	0	0	0	0	0	0	0	0	0	0	0	0	0
**des**	Sed			+	−	−	+	−	−	+	−	+	+	−	−	−	+	+	−	−	−	+	−	−
**Amours**	Swab			−	−	−				+	−	−	−	−	−	−	−	−	−	−	−	+	−	−
**Bain de**	Water	28,2	7	10	3	6	0	0	0	6	0	0	3	0	0	1	0	1	0	0	1	0	0	0
**Capès**	Sed						−	−	−	+	−	−	−	−	−	+	−	−	+	−	−	+	−	−
	Swab			−	−	−				−	−	−	−	−	−	−	−	−	−	−	−	−	−	−
**Bain de**	Water	31,7	8	0	0	0	0	0	2	0	0	0	0	0	0	0	0	2	0	0	2	0	0	0
**Dolé**	Sed																							
	Swab																							
**Dolé**	Water	31.9	8	4	12	0	0	0	0	0	2	4	0	0	0	0	0	7	1	0	0	0	0	0
**Amont**	Sed			−	+	−	+	+	−	−	−	−	+	+	+	+	−	+	+	−	−	+	+	+
	Swab			−	−	−	+	−	−	−	−	−	−	−	−	−	−	−	+	−	−	+	−	−
**Dolé**	Water	30,7	7,5	0	6	0				0	0	0	0	0	2	0	0	2	0	0	0	0	0	0
**Escalier**	Sed									+	−	−	−	−	−	−	−	−	−	−	−	−	−	−
	Swab			+	−	−				−	−	−	−	−	−	−	−	−	−	−	−	−	−	−

**Sp.:** thermophilic amoebae non-Naegleria sp., **N.l** : Naegleria lovaniensis., **N.f** : Naegleria fowleri,

(n) number of amoebae per liter of water; (+ ou -) presence or absence in sediment or swab; (blank) not investigated.

**Figure 2:**
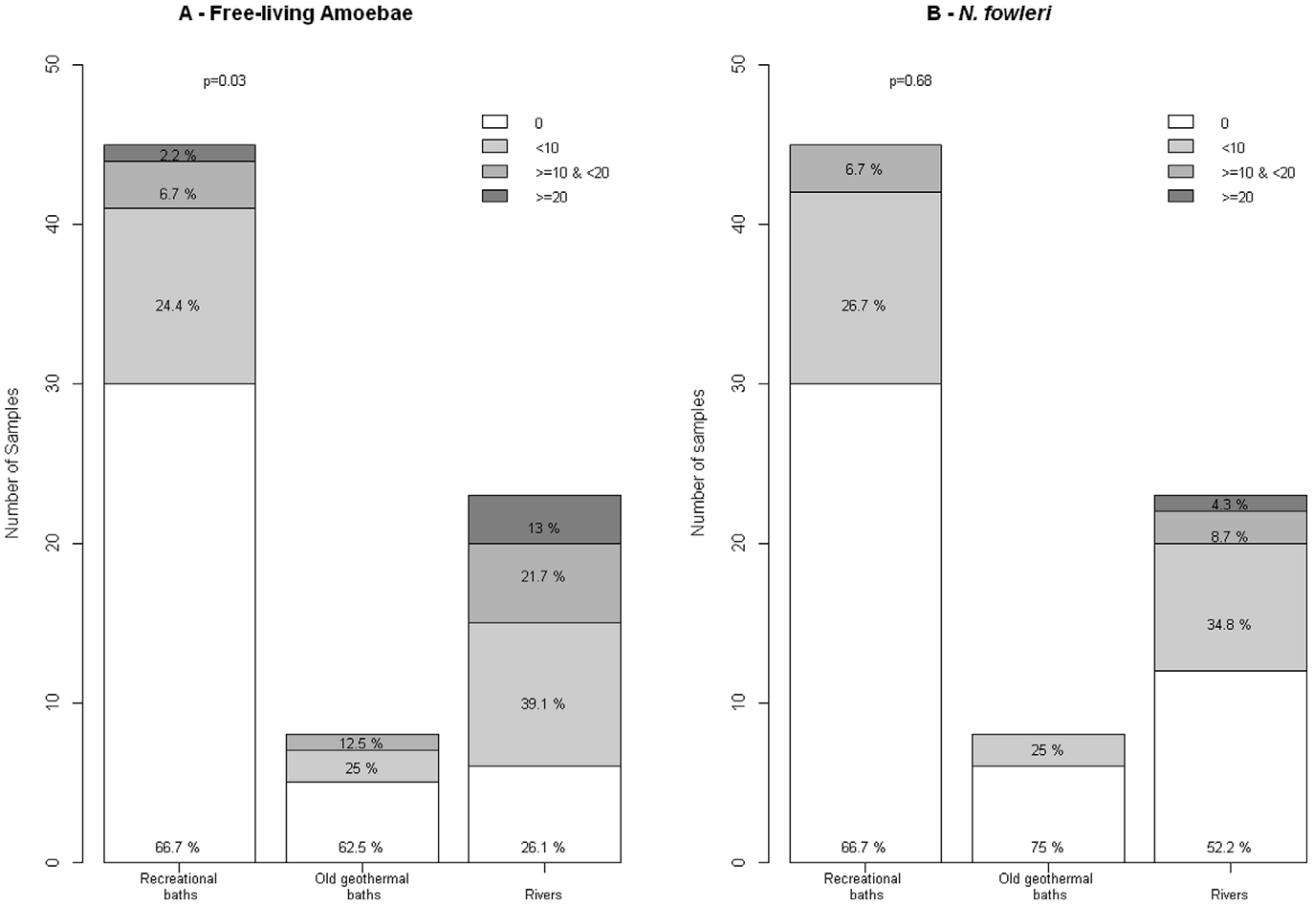
Levels of Free_living amoebae according to the type of water in Guadeloupe in 2011–2012. (A) free-living amoebae and (B) and *Naegleria fowleri*.

Differences were observed according to the type of waters analyzed. FLA were more often found and in higher concentrations in rivers (73.9% of positive samples), than in old geothermal baths (37.5%), recreational baths (33.3%) (p  = 0.03); the difference was not significant if we considered *N. fowleri* (Fig. 2).

The concentration of *N. fowleri* was rather low, from 0 to 22 amoebae per liter of water samples (Tables 1–2).

The most popular site of Bain de Dolé, yielded thermophilic amoebae, including *N. fowleri,* continually during the hot season between June and December in 2011, but less frequently and in lower concentration in 2012. Curiously, *N. fowleri* was less often isolated from the Bains Jaunes samples, although this bath is less frequently cleaned. Amoebae and in particular *N. fowleri* (1 per liter) were detected only once in 2011 in Bain des Amours but could be found sometimes in the sediments upstream of the bath in 2012.

**Figure 3:**
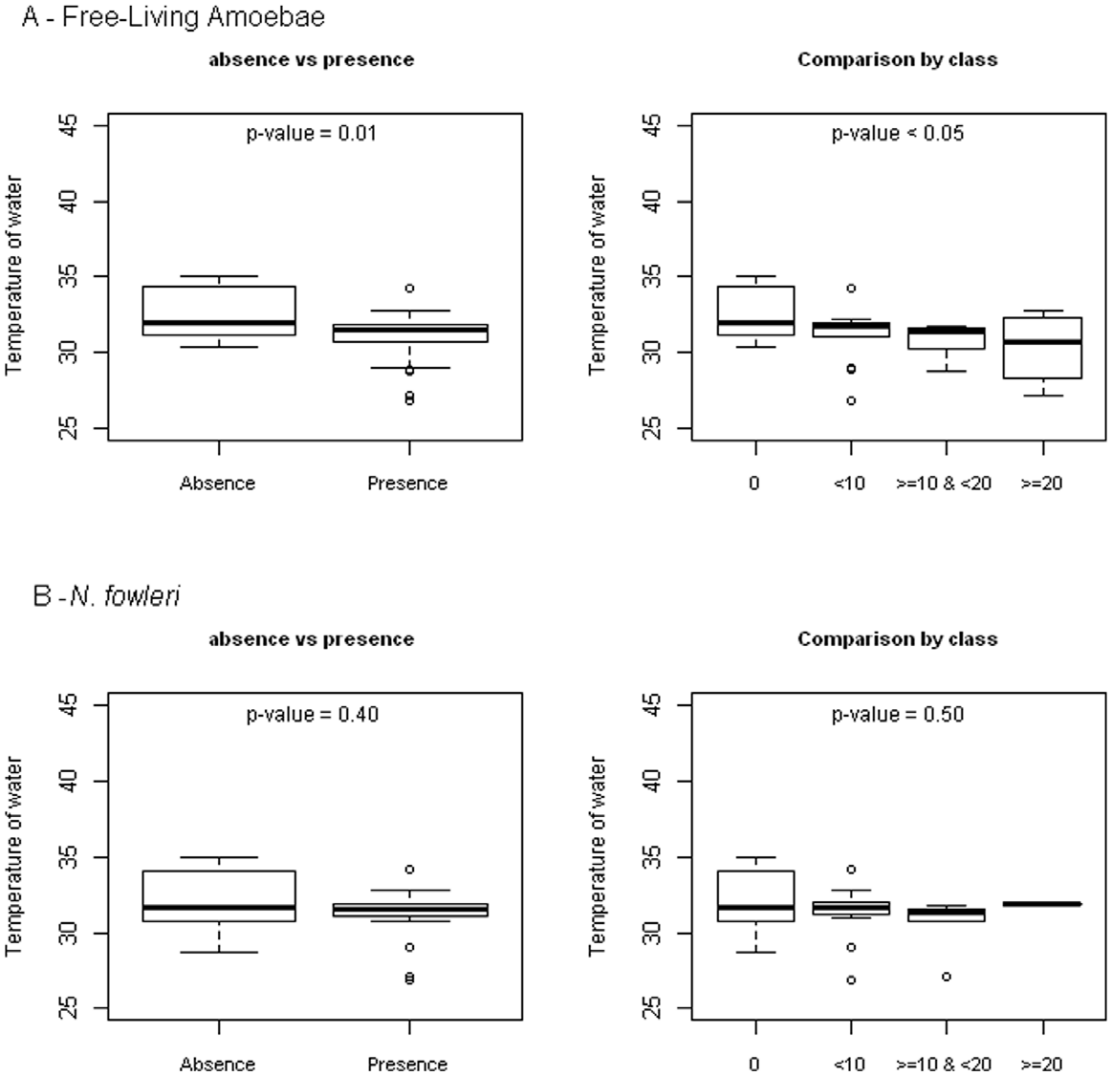
Levels of Free-living amoebae according to the temperature of water in Guadeloupe in 2011–2012. (A) free-living amoebae and (B) *Naegleria fowleri*.

Temperatures at the sites ranged from 26.9°C at Capes river to 34.9°C at Bains des Amours and the pH from 5.5 at Bains Jaunes to 9.0 at Dolé Amont (Table 3). No significant differences could be found in the presence or not of FLA or *N. fowleri* according to pH. Concerning temperature, FLA were significantly more frequently encountered (p<0.01) and in highest number (p<0.05) when the temperature decreased. No difference could be evidenced concerning *N. fowleri* (Fig. 3). These differences can also be due to the fact that amoeba were more numerous in the Capes River where the temperature was lower. No difference could be evidenced according to temperature in the same bath (Table 3).

**Table 3:** Number of *Naegleria fowleri* isolated from the most frequented baths of Guadeloupe according to temperature and pH (2011–12).

Dates	Bains Jaunes			Bain des Amours			Bain de Dolé			Dolé Amont			Bain de Capès		
	T°C	pH	*N.f*/L	T°C	pH	*N.f*/L	T°C	pH	*N.f*/L	T°C	pH	*N.f*/L	T°C	pH	*N.f*/L
**Jun-11**	31.7	5.5	0	33.6	6.9	0	31.4	8.2	8	ND	ND	ND	28.5	6.8	0
**Jul-11**	31.8	6.9	0	34.2	6.9	0	31.7	8.3	8	31.9	6.9	22	ND	ND	ND
**Aug-11**	30.7	6.5	0	34.2	7.0	2	31.9	8.2	7	31.0	6.9	0	ND	ND	ND
**Sep-11**	31.5	6.5	1	34.7	7.5	0	32.2	8.9	6	31.6	6.9	2	ND	ND	ND
**Oct-11**	31.8	6.3	5	34.7	7.6	0	31.5	8.9	2	31.6	9.0	6	31.5	7.9	6
**Nov-11**	31.6	6.7	10	34.4	7.6	0	31.7	8.8	13	31.5	8.2	2	ND	ND	ND
**Dec-11**	31.3	5.5	0	34.9	6.9	0	31.0	6.4	6	31.9	6.8	9	29.5	6.9	0
**Jan-12**	31.2	6.5	0	34.4	6.3	0	31.3	7.9	0	31.6	7.7	0	27.1	6.4	6
**Feb-12**	31.3	6.8	0	34.7	7.1	0	31.4	8.1	1	31.7	7.2	0	28.2	6.7	0
**Mar-12**	31.7	6.4	0	34.0	7.3	0	31.6	8.2	0	32.1	6.9	4	28.7	7.1	0
**Apr-12**	32.0	6.2	0	34.1	6.9	0	31.3	7.8	0	32.0	7.6	0	28.9	6.9	0
**May-12**	30.7	5.5	0	34.4	7.5	0	32.1	8.0	2	32.0	7.5	7	29.0	7.5	2
**Jun-12**	31.0	6.5	3	33.6	7.7	0	31.7	7.7	2	31.7	7.7	0	26.9	6.5	2
**Jul-12**	31.0	8.0	0	33.7	7.5	0	32.0	8.0	0	32.2	8.0	0	28.9	7.5	0

ND : non determined.

An analysis of physicochemical properties of water from the sampling places was performed (Table 4). Since parameters were rather stable in the same bath analyses were only performed from October 2011 to January 2012. Parameters could widely vary between sites, such as sulfates, which were found at a rather high concentrations in Bains Jaunes (around 315 mg/L) and at lower concentrations in other baths (from 51 mg/L in Bain de Capes to 92 mg/L in Bains des Amours). However, no correlation could be found between the concentration of the different parameters and the number of *N. fowleri,* with the exception of turbidity. Indeed, the number of *N. fowleri* increased along with turbidity (p  = 0.02).

**Table 4:** Number of *Naegleria fowleri* isolated from the most frequented baths of Guadeloupe according to physicochemical characteristics (2011–12).

Month	Baths	T°C	pH	*N.f*n/l	Turbidity NFU	Alkalimetric titre (°F)	Complete Alkalimetric titre (°F)	Total hardness (°F)	K mg/l	HCO mg/l	Ca mg/l	Mg mg/l	SO_4_ mg/l	Cl mg/l	Na mg/l	SiO_2_ mg/l	C mg/l	KMnO_4_ mg/l O_2_	NH_4_ mg/l	NO_3_ mg/l
	Bains Jaunes	31.8	6.4	5	0.3	0.0	2.0	22.1	6.0	24.4	95.0	23.7	310.0	59.0	37.6	112.5	<0.5	<0.4	<0.05	<1
	Bain de Capès	31.5	7.9	6	0.6	0.0	17.0	16.2	6.6	207.4	49.2	19.3	80.0	28.0	33.2	85.4	<0.5	0.8	<0.05	3.8
**Oct-11**	Bain des Amours	34.7	7.7	0	0.3	0.0	19.5	16.1	7.2	237.9	57.4	21.9	90.0	29.0	34.5	81.5	<0.5	0.9	<0.05	2.6
	Bain de Dolé	31.5	9.0	2	0.6	0.7	17.0	15.7	5.7	207.4	44.9	18.2	69.0	25.0	28.4	78.1	<0.5	<0.4	<0.05	3.1
	Dolé Amont	31.6	9.0	6	0.5	1.0	16.5	15.8	5.7	201.3	45.0	18.1	69.0	26.0	29.5	82.9	<0.5	0.9	<0.05	3.0
	Bains Jaunes	31.6	6.7	10	0.3	0.0	2.0	24.6	6.2	24.4	99.3	24.4	318.0	59.0	37.1	113.9	<0.5	<0.4	<0.05	<1
**Nov-11**	Bain des Amours	34.4	7.6	0	0.1	0.0	21.0	16.7	7.4	256.2	60.4	22.9	93.0	30.0	35.6	90.1	<0.5	<0.4	<0.05	2.8
	Bain de Dolé	31.7	8.8	13	0.4	0.0	17.0	16.4	5.8	207.4	48.5	18.7	69.0	25.0	30.1	76.7	<0.5	1.6	0.1	3.6
	Bains Jaunes	31.3	5.5	0	0.2	0.0	2.5	16.7	6.1	30.5	96.0	24.0	311.0	57.0	36.3	97.6	<0.5	<0.4	<0.05	<1
	Bain de Capès	29.5	6.9	0	0.1	0.0	15.0	14.0	5.1	184.2	37.9	147.1	51.0	21.0	25.5	68.4	<0.5	0.6	<0.05	6.4
**Dec-11**	Bain des Amours	34.9	6.9	0	0.2	0.0	21.0	20.0	7.2	256.2	57.1	23.0	92.0	29.0	34.6	80.1	0.9	1.2	<0.05	3.0
	Bain de Dolé	31.0	6.5	6	0.1	0.6	17.0	15.9	5.6	207.4	45.9	18.2	68.0	25.0	28.9	75.1	<0.5	<0.4	<0.05	3.8
	Dolé/Escalier	30.5	6.4	0	0.1	0.9	16.0	13.2	5.0	192.2	39.2	16.0	55.0	22.0	26.6	61.7	0.8	<0.4	<0.05	3.3
	Dolé/Amont	31.9	6.8	9	0.2	1.1	17.0	17.1	5.6	207.4	45.6	18.3	66.0	24.0	29.2	61.2	<0.5	1.6	<0.05	2.7
	Bains Jaunes	31.2	6.5	0	0.2	0.0	3.0	27.8	6.3	36.6	109.0	25.0	328.0	63.0	56.0	62.2	41.6	1.4	0.1	<1
**Jan-12**	Bain de Capès	27.1	6.4	6	0.9	0.0	14.5	13.1	5.2	176.9	42.0	15.0	56.0	22.0	27.0	62.6	0.6	1.8	<0.05	6.0
	Bain des Amours	34.4	6.4	0	0.4	0.0	21.0	19.6	7.4	256.2	63.0	23.0	96.0	30.0	36.0	52.5	0.9	0.1	<0.05	2.4
	Dolé/Escalier	30.2	6.5	0	0.1	0.6	16.0	14.5	5.1	195.2	48.0	18.0	66.0	23.0	27.0	58.8	<0.5	1.9	0.1	3.5

**Figure 4:**
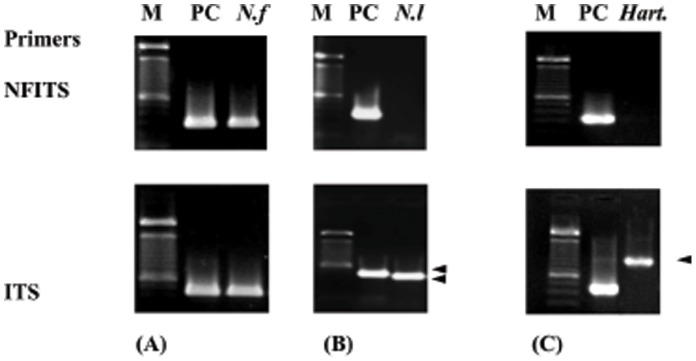
PCR results for the three major thermophilic amoeba species found in Guadeloupe. PCR detection and agarose gel electrophoresis of ITS rDNA of *N. fowleri* (A), *N. lovaniensis* (B) and *Hartmannella* sp**.** (C). **M :** 100 bp ladder, **PC** : Positive control (DNA of *Naegleria fowleri* (#. 359)) gives a single PCR product of 359 bp with the NFITS primers and a 448 bp with the ITS primer set. ***N.f*** : *Naegleria fowleri,* ***N.l*** : *Naegleria lovaniensis*, the ITS amplification gives a 403 bp PCR product while no band was obtained with the NFITS primers, ***Hart*** *.* : *Hartmannella* sp., the ITS amplification gives a 900 bp while no band was observed with the NFITS primer set.

The identifications by ITS sequencing gave relatively similar results in the different hot springs (Fig. 4). The only thermophilic amoebae detected and identified after culture at 44°C belonged to the genus *Naegleria* (*N. fowleri*, Fig. 4A and *N* *. lovaniensis* Fig. 4B) and to the genus *Hartmannella* (Fig. 4C). The latter, however, is indicated in databases to be *Vahlkampfia* [34], but a recent study [39] has demonstrated that the sequences of these AK-2007 strains do not belong to vahlkampfiids, but to the amoebozoan *Hartmannella.*

The *N. fowleri* DNA positive control (PC # 539) gives a PCR product of 448 bp with the ITS primer set and a 359 bp with NFITS primer set. *N. lovaniensis* was identified in samples in which an ITS-PCR fragment of 403 pb was obtained, while a NFITS-PCR fragment was absent. *Hartmannella sp*. was identified in samples in which an ITS-PCR fragment of 900 pb was obtained while a NFITS-PCR product was absent.

The distribution and identification of thermophilic amoebae in each sampling areas are shown in tables 1 and 2. The major ITS-PCR positive products obtained from amoebae isolated from the sampling sites were analyzed by DNA sequencing. Among a total of 41 amplicons that were sequenced, only 2 were obtained from thermophilic amoeba other than *Naegleria*. Gene sequences were submitted to the GenBank database in order to allow blast searching, and the highest percentage identity was taken to identify the species. The sequence analysis revealed that the pathogenic *N. fowleri* was the most frequently encountered thermophilic species, seconded by *N. lovaniensis;* in some cases *Harmannella* sp. was identified (see above about the *Vahlkampfia-Hartmannella* sp. identification). These sequencing results are shown in Table 5. *N. fowleri* was found with a 100% identity with the Genbank accession number # X96562.1 [37], in the following baths (Dolé, Dolé Amont, Bains Jaunes and Capes), while *N. lovaniensis* sequences (Dolé Amont, Dolé/Escalier and Capes) showed a 100% identity with the Genbank accession number # X96569.1 [37] (Table 6). Thermophilic *Hartmannella* sp. (indicated as *Vahlkampfia* sp. in databases, see above) with a 100% max identity with the Genbank accession number # AB330066.1 [34], were sequenced only in the bath of Capes (Table 6). The present study showed that all *N. fowleri* isolates from the Guadeloupean environment belonged to the Type-3 common Euro-American genotype with the 86 bp long ITS1 with a T instead of C at location 31 in the 5,8S rDNA [10], [37].

**Table 5:** ITS1 sequences of the three isolates identified in the different geothermal waters of Guadeloupe.

Acc. N°	ITS1 sequence	Isolate	Species
JX910445	atggtaaaaaaggtgaaaacctttttttatggtaaaaaaggtgtatggtaaaaaaggtgaaaaccttttttccatttacaaaaaat	DOLE	*N. fowleri*
JX910446	atggtaaaaaaggtgaaaaccttttttccttaattaaaaac	PIGEON	*N. lovanienesis*
JX910447	gaaccatcccggggagacaccaaaaaccgtagccccctcggcgaaacatcgatcgagggagggatcgctcgtcgatccttccgagaaacgagaat	CAPES	*Hartmanella* sp.

**Table 6:** Distribution of sequenced isolates from the different geothermal baths of Guadeloupe.

Acc. N°	Species	Bain de Capès	Bain de Dolé	Dolé Amont	Dolé Escalier	Bains Jaunes
JX910445	*N. fowleri*	**x**	**x**	**x**		**x**
JX910446	*N. lovaniensis*	**x**		**x**	**x**	
JX910447	*Hartmanella* sp.	**x**				

## Discussion

Our present study aimed at detecting the presence of pathogenic *N. fowleri* in the different recreational hot waters of Guadeloupe, without considering the exact numeration of amoebae in the water. At the beginning of 2011 we have tried different methods, such as different volumes of centrifugation since this method had been described as more efficient than filtration to recover trophozoites [40]. Due to the low number of amoeba detected, we have chosen filtration and to cut the filter in ten pieces because with the whole filter it is impossible to know if there is one amoeba or many. More accurate concentrations could probably be obtained by (i) determining the most probable number (MPN) per liter in serial repetitions of different sample volumes, either using different filtration membranes or by centrifuging the samples. However, when the concentrations are less than 100/l, it is difficult to have accurate numbers [40].

Taking into account the previously PAM case that occurred in 2008 and the high frequentation of the hot springs of Guadeloupe, we have chosen to focus the survey on the pathogenic *N. fowleri*. It is known that isolation of *N. fowleri* requires culture temperatures between 42°C and 45°C in an attempt to suppress the growth of other amoebae [5]. Accordingly, the culture temperature at 44°C used in this study was not suitable for the isolation of the other pathogenic *Naegleria* spp. which grow at 42°C maximum (*N. australiensis* and *N. italica*), and non-pathogenic and non-thermophilic *Naegleria* spp. Our results showed that the thermophilic and pathogenic *N. fowleri* is a common and widely distributed species in the hot waters in Guadeloupe. Considering the physical parameters, temperatures were almost similar throughout the year for the same place. On the other hand, pH could range from 5.5 to 9.0 in recreational baths, probably due to the cleaning procedure used or not in the bath. Indeed, Capes bath which is natural and never cleaned has a more narrow range of pH. Concerning the other physical and chemical parameters, given the stability of the results obtained from one month to another in each bath, between October 2011 and January 2012, period which included both rainy and dry periods, and due the high cost of analyses, we could not afford to carry these tests for a longer period. Only turbidity correlated with the number of amoebae. This can be explained by the fact that turbidity increases when sediments are suspended and it has been reported that concentrations of thermophilic *Naegleria* spp. and pathogenic *N. fowleri* can be higher in sediment samples [41]. In our study, sediments were less often positive than water samples (10.4% vs 38.3%). However, concentrations are probably much higher in sediments since we compared around 100 mg of sediments for each sample vs 500 g for water samples.

The public health ministry of France established an upper limit of 100 amoebae per liter, not to be exceeded in watercourses where human exposure is possible [42]. We found that the concentration of *N. fowleri* in the hot springs of Guadeloupe remained below this standard. However, our method probably underestimates the actual concentration [40], so that the concentration could sometimes be higher than 100 *N. fowleri* per liter in some waters. The detection of a PAM case related to these warm waters [19], could be due to the fact that human infection is possible with lower concentrations of *N. fowleri*. However the concentration may have been higher in 2008 since the baths were probably cleaned less often than now. Moreover, the dose of amoebae inhaled must be taken into account.

The other most frequently species is the thermophilic but non-pathogenic *N. lovaniensis*, which is also the closest phylogenetic relative [10]. It has been reported that *N. lovaniensis* is usually the most dominant species of *Naegleria* in warm water [5], but the present data show that in Guadeloupe *N. fowleri* is the most frequently encountered one. The reason for this feature observed in Guadeloupe should be the subject for further investigation.

In the recreational bath of Dolé, *N. fowleri* was found almost all year-round, in spite of a regularly cleaning of the bath, which suggests that there is a continuous upstream contamination of the water.

Pathogenic *N. fowleri* have been detected on all continents, except for Antarctica, and seven types were demonstrated to occur in Europe and three on the American continent [10]. Type 3 is a common type on both continents and was evidenced in all the hot waters tested in this study as well as in the previously described PAM case [19]. As depicted in the map showing the distribution of *N. fowleri* types and the possible routes of dispersal [10], the European Type 3 probably reached the Guadeloupean archipelago, located in the middle of the West Indies between the Caribbean sea and the North Atlantic ocean, from the American continent.

In summary, our study is the first to use a PCR-based approach to document the presence of *N. fowleri* in a variety of aquatic geothermal sites in the Guadeloupe National Park. Our results provide evidence that *N. fowleri* is not a transient organism but thrives in most hot springs. This amoeba poses health risks to people who use the hot springs for recreation. Obviously, considering the thousands of persons who swim each year in the different hot springs of Guadeloupe, the probability of being in contact with *N. fowleri* is rather important. This is in contrast with the only one case of PAM described in Guadeloupe, although it cannot be excluded that other cases escaped diagnosis. Considering the limited understanding of the ecology of *N. fowleri*, practical measures for prevention and control of *Naegleria* infections include education of the public, awareness within the medical community and regular survey of swimming areas. Our results allowed the regional health agency to develop a communication campaign and to install panels explaining the potential infestation of all the geothermal recreational waters of Guadeloupe.
